# GPT-4 Turbo with Vision fails to outperform text-only GPT-4 Turbo in the Japan Diagnostic Radiology Board Examination

**DOI:** 10.1007/s11604-024-01561-z

**Published:** 2024-05-11

**Authors:** Yuichiro Hirano, Shouhei Hanaoka, Takahiro Nakao, Soichiro Miki, Tomohiro Kikuchi, Yuta Nakamura, Yukihiro Nomura, Takeharu Yoshikawa, Osamu Abe

**Affiliations:** 1 Department of Radiology, The International University of Health and Welfare Narita Hospital, 852 Hatakeda, Narita, Chiba Japan; 2 Department of Computational Diagnostic Radiology and Preventive Medicine, the University of Tokyo Hospital, 7-3-1 Hongo, Bunkyo-Ku, Tokyo, Japan; 3 Department of Radiology, School of Medicine, Jichi Medical University, 3311-1 Yakushiji, Shimotsuke, Tochigi Japan; 4 Center for Frontier Medical Engineering, Chiba University, 1-33 Yayoicho, Inage-Ku, Chiba, Japan; 5 Department of Radiology, the University of Tokyo Hospital, 7-3-1 Hongo, Bunkyo-Ku, Tokyo, Japan

**Keywords:** Artificial intelligence (AI), Large language model (LLM), ChatGPT, GPT-4 Turbo, GPT-4 Turbo with Vision, Japan Diagnostic Radiology Board Examination (JDRBE)

## Abstract

**Purpose:**

To assess the performance of GPT-4 Turbo with Vision (GPT-4TV), OpenAI’s latest multimodal large language model, by comparing its ability to process both text and image inputs with that of the text-only GPT-4 Turbo (GPT-4 T) in the context of the Japan Diagnostic Radiology Board Examination (JDRBE).

**Materials and methods:**

The dataset comprised questions from JDRBE 2021 and 2023. A total of six board-certified diagnostic radiologists discussed the questions and provided ground-truth answers by consulting relevant literature as necessary. The following questions were excluded: those lacking associated images, those with no unanimous agreement on answers, and those including images rejected by the OpenAI application programming interface. The inputs for GPT-4TV included both text and images, whereas those for GPT-4 T were entirely text. Both models were deployed on the dataset, and their performance was compared using McNemar’s exact test. The radiological credibility of the responses was assessed by two diagnostic radiologists through the assignment of legitimacy scores on a five-point Likert scale. These scores were subsequently used to compare model performance using Wilcoxon's signed-rank test.

**Results:**

The dataset comprised 139 questions. GPT-4TV correctly answered 62 questions (45%), whereas GPT-4 T correctly answered 57 questions (41%). A statistical analysis found no significant performance difference between the two models (P = 0.44). The GPT-4TV responses received significantly lower legitimacy scores from both radiologists than the GPT-4 T responses.

**Conclusion:**

No significant enhancement in accuracy was observed when using GPT-4TV with image input compared with that of using text-only GPT-4 T for JDRBE questions.

## Introduction

Recent advancements in large language models (LLMs) have marked a significant evolution in the field of artificial intelligence (AI). Among the numerous LLM-based applications, ChatGPT, which is based on the generative pre-trained transformer (GPT) architecture, has gained widespread recognition for its extensive capabilities [1–3]. Although not specifically designed for medical applications, ChatGPT possesses a substantial repository of medical knowledge, enabling it to handle healthcare-related queries. Kung et al. reported that ChatGPT, powered by the GPT-3.5 model, attained scores above or close to passing thresholds in the United States Medical Licensing Examination (USMLE) [4]. More recent studies have indicated that the latest GPT-4 model successfully attained passing scores in medical licensing examinations in several countries including Japan, China, Poland, and Peru [5–10]. Several studies have evaluated the performance of ChatGPT in radiology. Bhayana et al. reported that GPT-3.5 nearly passed a radiology-board-style examination that resembles the Canadian Royal College and American Board of Radiology examinations [11]. Toyama et al. reported that the GPT-4 scored slightly above the provisional passing limit when applied to questions from the Japan Radiology Board Examination [12]. However, because ChatGPT was originally unable to accept image inputs, questions necessitating the interpretation of input images were not included in these studies.

The introduction of GPT-4 V(ision), an advanced iteration of GPT-4 featuring image processing capabilities, marks a significant leap beyond the initial text-only functionality of ChatGPT [3]. This version processes and interprets images in conjunction with textual data, broadening its applicability to fields that require image analysis. Yang et al. reported that the accuracy of GPT-4 improved from 83.6 to 90.7% when images were provided as input along with text for the USMLE [13]. Notably, the images utilized in the study were primarily non-radiological visuals, such as photographic images, pathological slides, electrocardiograms, and diagrams. Consequently, the diagnostic capabilities of GPT-4 V on radiological images, especially in challenging tasks, remain unexplored. Enhancing GPT-4 V to achieve a high diagnostic accuracy in interpreting radiological images may offer significant benefits to both diagnostic radiologists and physicians in clinical practice. This study was conducted to evaluate the diagnostic accuracy of GPT-4 Turbo with Vision (GPT-4TV)—the latest iteration of GPT-4 V—and compare it with that of its text-only counterpart, GPT-4 Turbo (GPT-4 T), in the Japan Diagnostic Radiology Board Examination (JDRBE) that assesses extensive expertise in diagnostic radiology. Our larger objective was to determine the impact of integrating visual data on the performance of AI models in diagnostic radiology.

## Materials and methods

### Study design

This retrospective study did not directly involve human subjects. All data used in this study have been anonymized and are devoid of any information that could identify individuals, and these are available online to all Japan Radiology Society (JRS) members. Furthermore, all data have been input through the OpenAI application programming interface (API), as explained in subsequent sections. OpenAI ensures that data submitted via the API are encrypted, securely retained with strict access controls, deleted from the systems after 30 days, and not used for model training [14]. Therefore, approval from the Institutional Review Board was waived.

### Questions dataset

All questions used in our experiments were sourced from the JDRBE, which assesses in-depth knowledge of diagnostic radiology. To be eligible for the JDRBE, candidates must initially acquire the Japan Radiology Specialist certification, which involves completing a minimum of a 3-year training program and passing the Japan Radiology Board Examination. Furthermore, an additional 2-year training period in diagnostic radiology is mandatory for JDRBE eligibility.

**Fig. 1:**
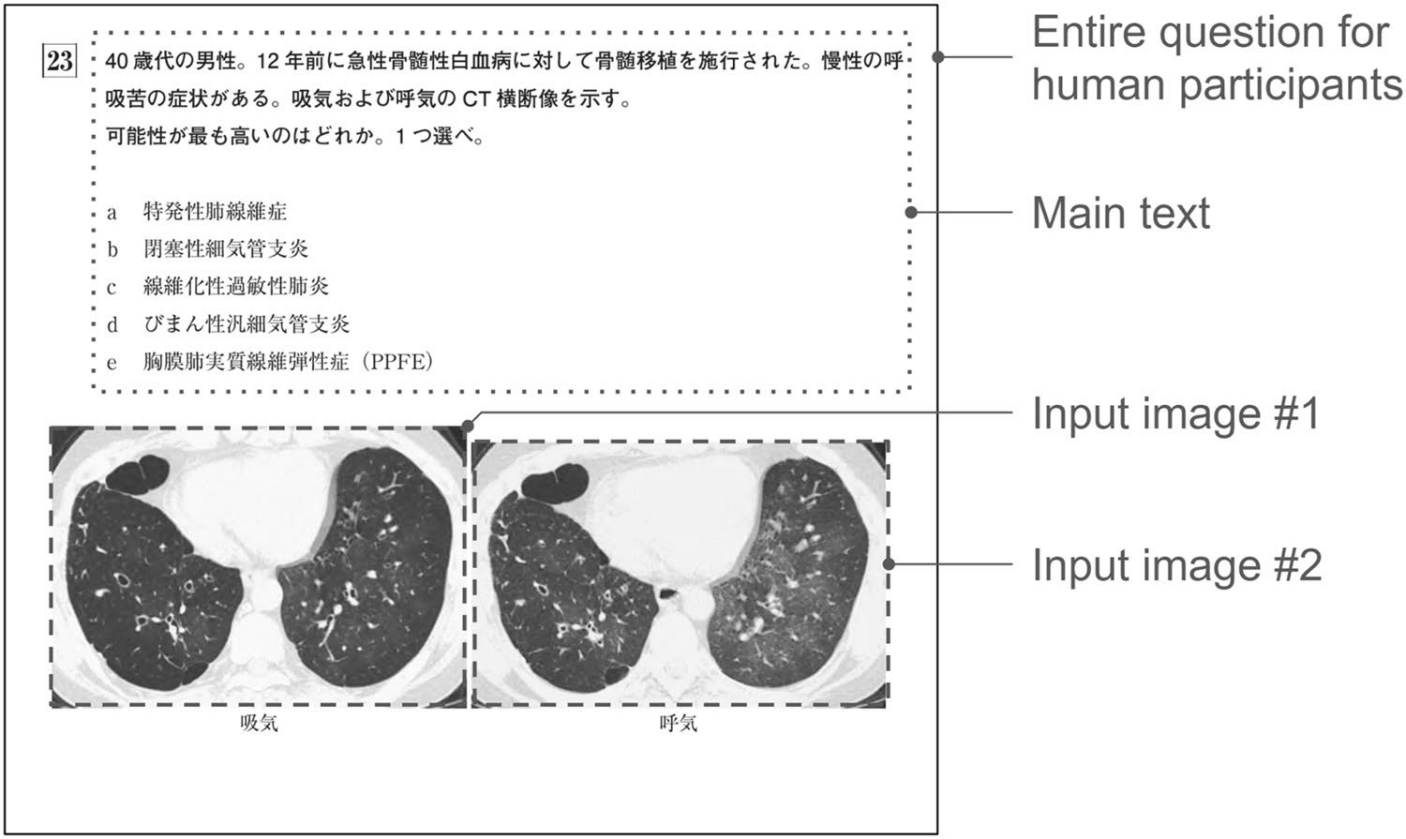
Example of text and image extraction from a question. The main text and input images were extracted and fed to the models. The question number (“23”) and image captions (“inhalation” and “exhalation”) were omitted from the input. In this question, the main text states that “axial CT images during inhalation and exhalation are shown”

The examination papers are exclusively accessible to JRS members via the website. Each paper was originally provided in the Portable Document Format (PDF). To extract texts and images, we converted the PDF files into the eXtensive Markup Language (XML) format using Adobe Acrobat (Adobe, San Jose, California, US). All extracted images retained their original resolutions, and were in PNG or JPEG format. Heights ranged from 134 to 1708 pixels (mean: 447), and widths ranged from 143 to 950 pixels (mean: 474). For the extracted texts, we only used the main text from each question; any other texts, including the captions of input images, were discarded. Note that as the problem statement details each input image, an understanding of what each image represents is conveyed even in the absence of captions. Figure 1 exemplifies a question extracted in this way.

Our dataset consisted of questions from the JDRBE 2021 and 2023. Questions from JDRBE 2022 were not included because we failed to extract the input data due to inconsistency in the provided PDF file. Questions without any accompanying images were excluded, and each of the remaining questions was accompanied by one to four images. Each question had five possible choices, and approximately 90% of the questions were of the single-answer type, requiring the selection of one correct option out of five. The remaining 10% were two-answer questions, wherein participants had to choose two correct options from the five available, and a response was deemed correct only if both options were correct. The required number of correct options was specified in the text.

Because the answers were not officially published, six board-certified diagnostic radiologists (Y.N., T.K., T.N., S.M., S.H., and T.Y.; with 6, 7, 8, 16, 21, and 28 years of experience in diagnostic radiology, respectively) determined the ground-truth answers, consulting relevant literature as necessary. Three or more radiologists were assigned to each question, and consensus was reached through discussions. Questions without unanimous agreement on answers were excluded. All questions were classified into the following 11 subspecialties: breast, cardiovascular, gastrointestinal, genitourinary, head and neck, musculoskeletal, neuroradiology, pediatric, thoracic, interventional radiology, and nuclear medicine.

**Fig. 2:**
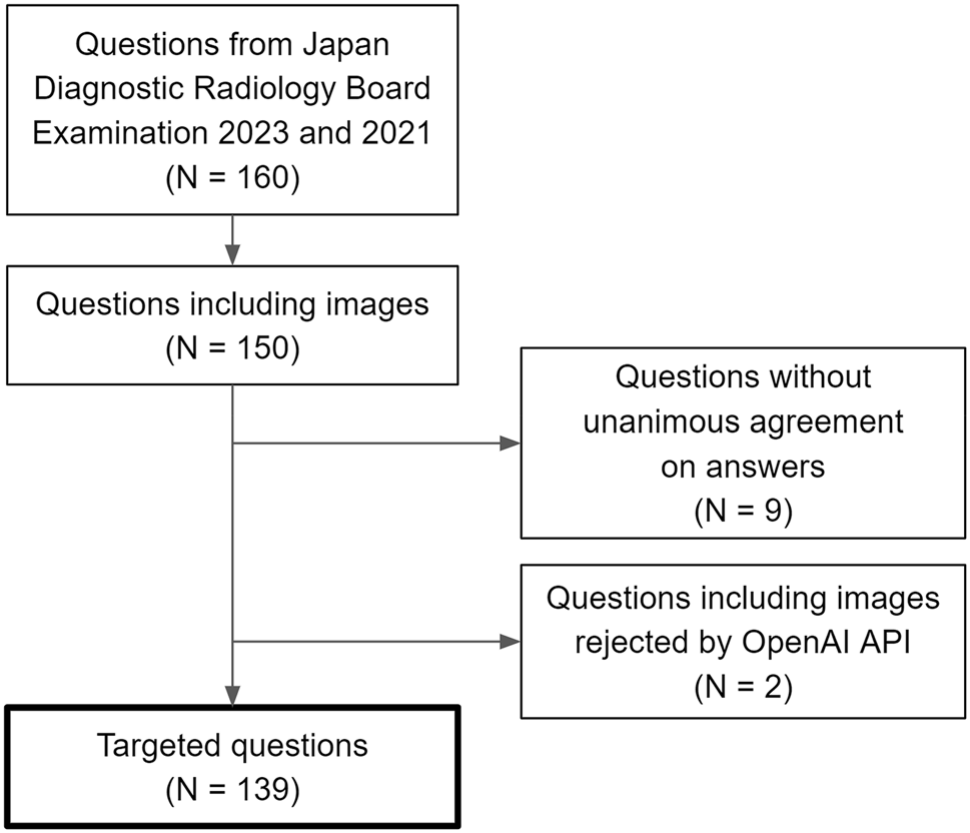
Summary of questions included in this study

During the experiments, we further excluded two questions from the dataset due to the rejection of their corresponding images by the OpenAI API, which flagged them for potentially containing unsafe content. Figure 2 illustrates a flow chart detailing the inclusion and exclusion processes for questions.

### Experimental details

We evaluated the performance of GPT-4TV and GPT-4 T on the prepared dataset. Because GPT-4TV is designed to accept visual input, it was passed both text and image data from the questions, whereas GPT-4 T solely received textual input without any accompanying images. Questions were submitted through the OpenAI API. The internal version of GPT-4TV was *gpt-4-vision-preview*, whereas that of GPT-4 T was *gpt-4–1106-preview*. Both models were released on November 2023 and trained on data dating up to April 2023 [15]. We set the *max_tokens* parameter of the API to the maximum of 4,096, and used the default settings for all other parameters. Because all questions from the examinations were in Japanese, the textual data were passed to the models without translation. We provided a shared prompt in Japanese along with the questions, as shown in Table 1. The prompt included the instruction, “Even if you are not confident, you will always be forced to select and provide an answer,” ensuring that the models answer as many questions as possible. In cases where the models initially refused to provide an answer, or if an error occurred during the experiment, we re-entered the same prompt and question until we successfully received an answer. All experiments were conducted between January 16–17, 2024.

**Table 1:** Prompts used in the experiments

	Prompt in Japanese	English translation
GPT-4TV	あなたはこれから放射線科診断専門医試験を受験する放射線科医です。以下に専門医試験の問題を提示しますので、解答をお願いします。また、解答に至った思考過程も簡潔に説明してください。自信がない場合でも必ず解答を選択して答えることが強制されます。	You are a radiologist who is about to take the Japan Diagnostic Radiology Board Examination. Below we present a question for the examination and ask you to answer it. Please also briefly explain the thought process that led you to your answer. Even if you are not confident, you will always be forced to select and provide an answer
GPT-4 T	あなたはこれから放射線科診断専門医試験を受験する放射線科医です。以下に専門医試験の問題を提示しますので、解答をお願いします。また、解答に至った思考過程も簡潔に説明してください。ただし、問題文のみで、画像は与えられません。自信がない場合でも必ず解答を選択して答えることが強制されます。	You are a radiologist who is about to take the Japan Diagnostic Radiology Board Examination. Below we present a question for the examination and ask you to answer it. Please also briefly explain the thought process that led you to your answer. Note that you will be given only the text of the questions, without any images. Even if you are not confident, you will always be forced to select and provide an answer

To assess the radiological credibility of responses generated by GPT-4TV and GPT-4 T, two diagnostic radiologists, Y.H. and S.M., with 2 and 16 years of diagnostic radiology experience, respectively (S.M. is board-certified), independently assigned legitimacy scores using a five-point Likert scale (5 = Excellent, 4 = Good, 3 = Fair, 2 = Poor, and 1 = Very poor). The radiologists were blinded to each other’s assessments, and all responses were presented in a random order. Both radiologists were informed of the model (GPT-4TV or GPT-4 T) associated with each response. Legitimacy scores were rated subjectively based on how reasonable the response was according to the information provided to each model (i.e., for GPT-4 T, a response was considered excellent if it made a reasonable guess from what could be determined solely from the textual information). The quadratic weighted kappa coefficient [16] was calculated to measure the degree of mutual agreement between the two raters.

### Statistical analysis

Differences in performance between GPT-4TV and GPT-4 T were analyzed using McNemar’s exact test, with subgroup analyses conducted for single- and two-answer questions. Differences in legitimacy scores between GPT-4TV and GPT-4 T were analyzed using Wilcoxon’s signed-rank test. Statistical significance was set at P < 0.05. All analyses were conducted using the Python software (version 3.11.4) along with SciPy (version 1.12.0) and statsmodels (version 0.14.1) libraries.

## Results

**Fig. 3:**
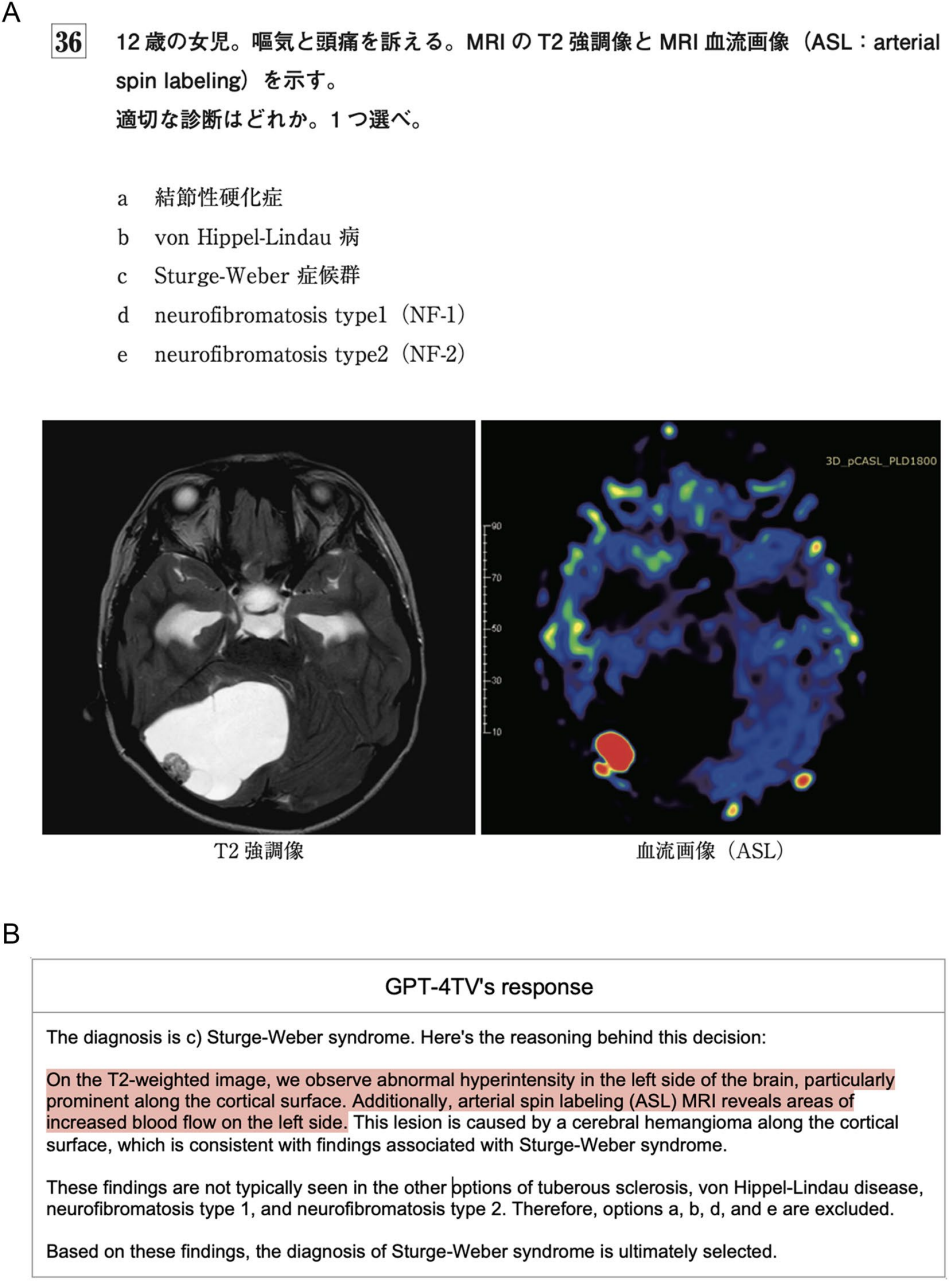

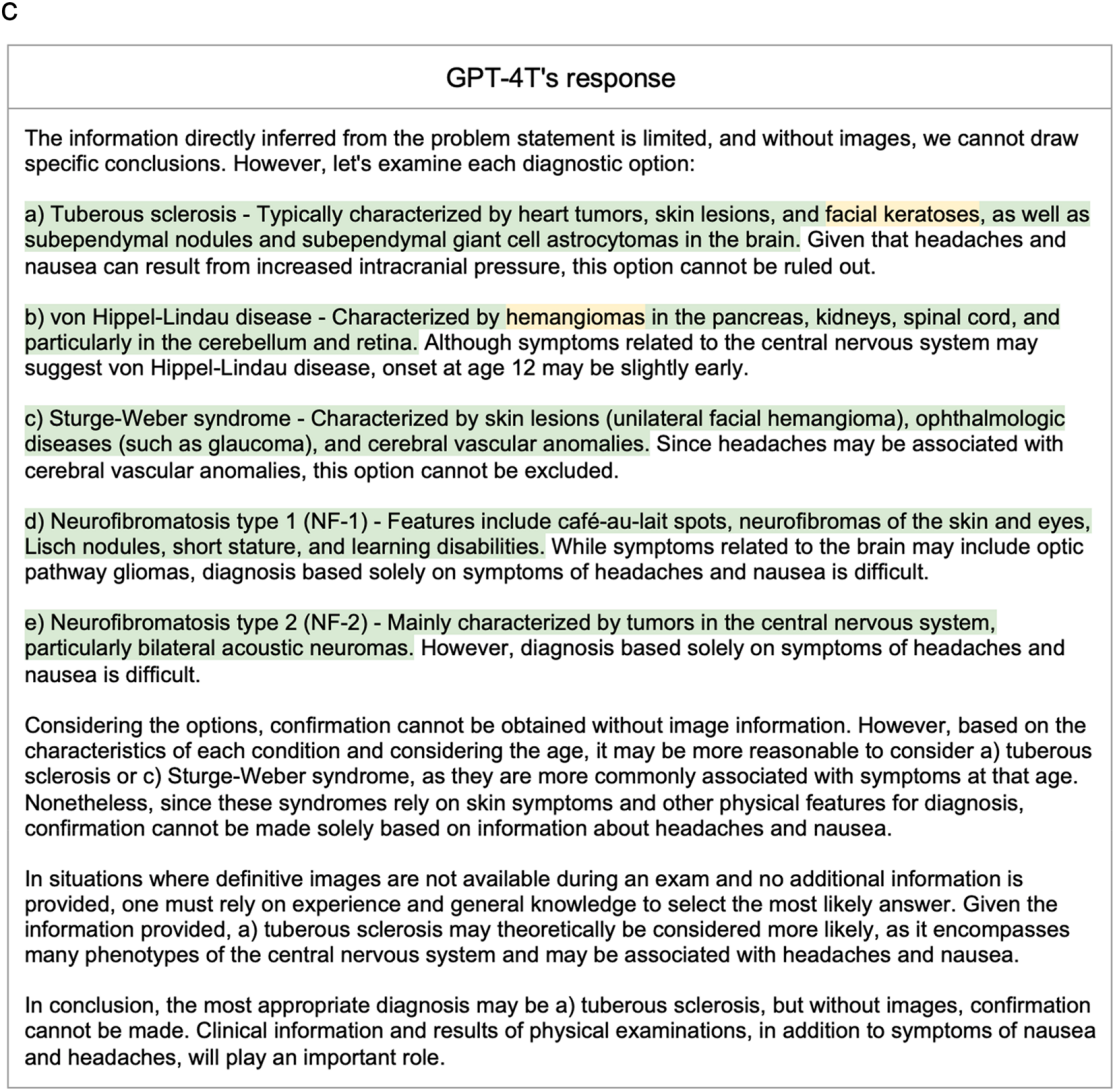
**a** Question 36 from the Japan Diagnostic Radiology Board Examination 2021, representing a clinical scenario of a 12-year-old girl with complaints of nausea and headaches. The question asks to identify the most probable diagnosis from the following options: (a) tuberous sclerosis, (b) von Hippel-Lindau disease (vHL), (c) Sturge-Weber syndrome, (d) neurofibromatosis type 1, and (e) neurofibromatosis type 2. Axial MRI scans of T2-weighted image and arterial spin labeling image are included. The correct answer is (b) vHL. **b** GPT-4TV’s response to the question presented in Fig. 3a, translated into English. Text highlighted in red indicates inaccurate image interpretation. **c** GPT-4 T’s response to the question presented in Fig. 3a, translated into English. Text highlighted in green indicates medically accurate descriptions of the provided options. Text highlighted in yellow indicates terminology that is not strictly accurate

The dataset encompassed 139 questions. Table 2 displays the frequency of modalities and planes across all questions in the dataset. Figure 3 illustrates an example question along with corresponding responses (translated into English by us) from GPT-4TV and GPT-4 T. Table 3 lists the performance metrics achieved by GPT-4TV and GPT-4 T for the dataset. GPT-4TV achieved a correct answer rate of 45% (62 out of 139 questions), whereas GPT-4 T achieved a correct answer rate of 41% (57 out of 139 questions). The difference in accuracy between the two conditions was not statistically significant (P = 0.44). The two models selected the same option(s) for 86 questions (62%). In the subgroup analyses, no significant difference in accuracy between GPT-4TV and GPT-4 T was observed for the single- and two-answer cohorts. Table 4 presents a contingency table describing the numbers of correct and incorrect answers from GPT-4TV and GPT-4 T.

**Table 2:** Details of the questions used

	No. of questions	No. of images
Total	139	290
Average number of images		2.1
Modalities		
CT	65	115
Axial	62	95
Coronal	12	12
Sagittal	4	4
Other	3	4
MRI	51	96
Axial	33	54
Coronal	9	13
Sagittal	11	17
Other	8	12
Nuclear medicine	30	56
Other	20	23

For modalities, each value of “No. of questions” represents the number of questions with at least one associated image of that modality (and plane, if specified)

**Table 3:** Performance comparison between GPT-4TV and GPT-4 T

	No. of questions	GPT-4TV	GPT-4 T	P-value
All questions	139	62 (45%)	57 (41%)	0.44
Single-answer questions	123	55 (45%)	50 (41%)	0.44
Two-answer questions	16	7 (44%)	7 (44%)	1.0
Subspecialty				
Breast	6	2 (33%)	3 (50%)	
Cardiovascular	9	2 (22%)	2 (22%)	
Gastrointestinal	22	8 (36%)	4 (18%)	
Genitourinary	11	3 (27%)	4 (36%)	
Head and Neck	5	3 (60%)	2 (40%)	
Musculoskeletal	11	4 (36%)	2 (18%)	
Neuroradiology	15	8 (53%)	7 (47%)	
Pediatric	6	2 (33%)	2 (33%)	
Thoracic	20	12 (60%)	11 (55%)	
Interventional radiology	4	3 (75%)	3 (75%)	
Nuclear medicine	30	15 (50%)	17 (57%)

**Table 4:** Numbers of correct and incorrect responses from GPT-4TV and GPT-4 T

No. of questions		GPT-4TV		
		Correct	Incorrect	Total
GPT-4 T	Correct	46 (33%)	11 (8%)	57 (41%)
	Incorrect	16 (12%)	66 (47%)	82 (59%)
	Total	62 (45%)	77 (55%)	139 (100%)

Table 5 shows the distribution of legitimacy scores for GPT-4TV and GPT-4 T responses. The quadratic weighted kappa coefficient between the two raters was 0.517, indicating moderate agreement [17]. Both raters provided significantly lower legitimacy scores for GPT-4TV responses than those for GPT-4 T responses.

**Table 5:** Distribution of legitimacy scores for responses generated by GPT-4TV and GPT-4 T

Score	Rater #1 (Y.H.)		Rater #2 (S.M.)	
	GPT-4TV	GPT-4 T	GPT-4TV	GPT-4 T
1 (Very poor)	32	0	4	1
2 (Poor)	24	3	37	11
3 (Fair)	38	6	29	29
4 (Good)	19	19	26	53
5 (Excellent)	26	111	43	45
Median	3	5	3	4
*P* value	< 0.001		< 0.001	

## Discussion

In our investigation, we compared the performance of GPT-4TV with that of GPT-4 T on questions from the JDRBE. We found no statistically significant difference in accuracy between the two models (Table 3). Moreover, the two models selected the same option(s) for a substantial portion of the questions (62%). These results suggest that GPT-4TV primarily depends on linguistic cues for decision-making, with images playing a supplementary role.

As shown in Table 5, GPT-4 T received exceptionally high legitimacy scores (medians of 5 and 4) in subjective analysis, partly because it accurately recalled diseases (e.g. autosomal dominant polycystic kidney disease, multiple endocrine neoplasia, Birt-Hogg-Dubé syndrome) from patient characteristics and options, even without images. Despite the absence of image data, GPT-4 T often selected the most plausible option based on epidemiological knowledge, contributing to its high subjective scores. In contrast, GPT-4TV received significantly lower subjective scores, primarily stemming from numerous image interpretation errors, including ones that are considered basic by radiologists (e.g. mislabeling hyperintensity as hypointensity, misidentifying lesion locations including laterality). Because the evaluators were specialized radiologists, they were potentially more likely to note image interpretation errors, leading to a more critical evaluation.

In Fig. 3, both GPT-4TV and GPT-4 T selected an incorrect option for the given question. GPT-4 T carefully assessed the likelihood of each option solely based on the clinical information provided in the problem statement and identified tuberous sclerosis as the most probable diagnosis. Though this conclusion was incorrect, it received legitimacy scores of 5 (Excellent) and 3 (Fair) from the raters. Conversely, the image interpretation by GPT-4TV was highly inaccurate, resulting in a legitimacy score of 1 (Very poor) from both raters. This case underscores the limited proficiency of GPT-4TV in image interpretation and its negative impact on the subjective impressions of radiologists, despite both models having arrived at an incorrect conclusion.

Although GPT-4TV received significantly lower legitimacy scores with addition of input images, the final accuracy rate did not decrease. This may be because, as mentioned earlier, ChatGPT does not place much emphasis on image information. The nature of prioritizing linguistic information over image information does not align well with the responsibilities of radiologists. If this is a common characteristic of LLMs, it could pose a barrier when constructing general-purpose image diagnostic AI systems to assist radiologists.

The ability of multimodal GPT models to interpret medical images is an active research area. Yang et al. [13] reported a notable improvement in GPT-4’s performance on USMLE questions with the addition of images to supplement text inputs. In contrast, our previous study [18] demonstrated that GPT-4 V did not significantly outperform the text-only GPT-4 in answering questions from the Japanese National Medical Licensing Examination. Our present findings align with this, as we observed no significant enhanced accuracy in GPT-4TV compared to GPT-4 T. This variability in performance improvement suggests that the efficacy of integrating both text and image inputs, as opposed to relying solely on text, may vary depending on the nature of the input question. Notably, the images used in previous studies largely represented non-radiological visuals including photographic images, pathological slides, electrocardiograms, and diagrams. In contrast, the present study predominantly focused on radiological images, particularly CT and MRI, as well as nuclear medicine imagery. Furthermore, the JDRBE targets board-certified radiologists with a minimum of five years of radiological experience, making it more challenging than the examinations utilized in prior studies. These differences in the data may account for the observed variations in accuracy improvement. Another key difference is the language of the input texts: Yang et al. used English, whereas our studies used Japanese. Although the input language has been noted to affect GPT model performance [3], the extent of this impact in our studies remains unclear. Future research should explore how input languages influence performance, perhaps by comparing the outcomes between Japanese texts provided as is and those translated into English.

This study has several limitations. First, the inherent generative nature of ChatGPT can result in different outputs for identical prompts and questions, which may have affected study outcomes. Notably, we recorded only a single response from each model per question without examining the potential variability in responses. Ideally, a more extensive analysis must be conducted to investigate the extent of this variability; however, this aspect was not explored in our study. Second, the training data for GPT-4TV and GPT-4 T were dated up to April 2023, which was more recent than the online disclosure of examination papers. Although access to examination data is restricted to JRS members, some of the questions may have been included in ChatGPT’s training dataset through various scenarios, such as if a JRS member had input the questions into ChatGPT’s web interface or inadvertently published them online before April 2023. Third, our experiments employed only one prompt per model, potentially overlooking more effective prompts. Lastly, the raters were aware of the model (GPT-4TV or GPT-4 T) linked to each response, which may have introduced cognitive bias.

In conclusion, this study found no notable benefit in employing GPT-4TV with image inputs to respond to JDRBE questions compared with that of using GPT-4 T solely with text. The outcomes of this study underscore the need for future research to explore more sophisticated methodologies for multimodal models, particularly in challenging domains such as those exemplified by the JDRBE.

## Appendix

### Questions where only one of the two models was correct

Table 6 lists detailed information pertaining to the 27 questions for which only one of the models was correct. Among these, four gastrointestinal questions received correct responses from GPT-4TV and incorrect responses from GPT-4 T, whereas no gastrointestinal questions received incorrect responses from GPT-4TV and correct responses from GPT-4 T. This led to an 18% discrepancy in accuracy on gastrointestinal questions between GPT-4TV and GPT-4 T, as shown in Table 3. It is possible that GPT-4TV is slightly more accurate in answering gastrointestinal questions. Apart from this, there was no observable trend regarding the difference in modalities or subspecialties between the two groups.

**Table 6:** Questions where (A) only GPT-4TV was correct and (B) only GPT-4 T was correct

No. of questions	16	No. of total input images	33
(A) Correct responses from GPT-4TV and incorrect responses from GPT-4 T			
Single-answer questions	16	CT	13
Two-answer questions	0	Axial	12
		Coronal	1
Subspecialty		Sagittal	0
Gastrointestinal	4	Other	0
Neuroradiology	3	MRI	13
Thoracic	3	Axial	8
Head and Neck	2	Coronal	2
Musculoskeletal	2	Sagittal	2
Nuclear medicine	2	Other	1
		Nuclear medicine	3
		Other	4

No. of questions	11	No. of total input images	28
(B) Incorrect responses from GPT-4TV and correct responses from GPT-4 T			
Single-answer questions	11	CT	10
Two-answer questions	0	Axial	10
		Coronal	0
Subspecialty		Sagittal	0
Nuclear medicine	4	Other	0
Neuroradiology	2	MRI	11
Thoracic	2	Axial	5
Breast	1	Coronal	2
Genitourinary	1	Sagittal	4
Head and Neck	1	Other	0
		Nuclear medicine	7
		Other	0
